# Kinome-Wide Screening with Small Interfering RNA Identified Polo-like Kinase 1 as a Key Regulator of Proliferation in Oral Cancer Cells

**DOI:** 10.3390/cancers11081117

**Published:** 2019-08-05

**Authors:** Yih-Gang Goan, Pei-Feng Liu, Hsueh-Wei Chang, Hung-Chih Chen, Wen-Chi Chen, Shyh-Ming Kuo, Cheng-Hsin Lee, Chih-Wen Shu

**Affiliations:** 1 Department of Surgery, Kaohsiung Veterans General Hospital Pingtung Branch, Pingtung 91245, Taiwan; 2 Department of Nursing, Meiho University, Pingtung 91202, Taiwan; 3 Department of Biomedical Science and Environmental Biology, Kaohsiung Medical University, Kaohsiung 80708, Taiwan; 4 Cancer Center, Kaohsiung Medical University Hospital, Kaohsiung Medical University, Kaohsiung 80708, Taiwan; 5 Center for Cancer Research, Kaohsiung Medical University, Kaohsiung 80708, Taiwan; 6 Division of Oral & Maxillary Surgery, Department of Stomatology, Kaohsiung Veterans General Hospital, Kaohsiung 81362, Taiwan; 7 Division of Gastroenterology and Hepatology, Department of Internal Medicine, Kaohsiung Veterans General Hospital, Kaohsiung 81362, Taiwan; 8 School of Medicine, National Yang-Ming University, Taipei 11221, Taiwan; 9 Department of Biomedical Engineering, I-Shou University, Kaohsiung 82445, Taiwan; 10 School of Medicine for International Students, I-Shou University, Kaohsiung 82445, Taiwan

**Keywords:** kinome, siRNA screening, *PLK1*, oral squamous cell carcinoma, prognosis, biomarker

## Abstract

Oral squamous cell carcinoma (OSCC) is one of the major leading causes of cancer-related death worldwide, with limited effective markers for diagnosis and therapy, which has caused a low overall survival rate in the past decades. Kinases play important roles in tumor development and malignancy in various types of cancer. However, little is known about the role of kinases in OSCC cells. In this study, an arrayed kinome small interfering RNA (siRNA) library was used to screen oral cancer cell lines and counter assayed with normal fibroblast cells to identify the genes required for cancer cell proliferation. We found that polo-like kinase 1 (*PLK1*) was one of the most potent genes required for OSCC cell proliferation. The knockdown of *PLK1* with a siRNA or antisense oligonucleotide (ASO) consistently diminished cyclin-B1 (CCNB1) expression/phosphorylation and the G_2_-M phase transition. Similar effects were observed in cells treated with the PLK1 kinase inhibitor BI6727. Besides, The Cancer Genome Atlas (TCGA) analysis revealed that *PLK1* was elevated in tumor tissues and associated with short survival in patients with OSCC. We also found that *PLK1* expression was highly correlated with the expression of its downstream effector, CCNB1, in patients with OSCC. Coexpression of the two genes resulted in a poor prognosis of OSCC patients, particularly those in the advanced stages of OSCC. Taken together, our results suggest that *PLK1* might be a diagnostic or therapeutic marker for OSCC.

## 1. Introduction

Oral squamous cell carcinoma (OSCC) is one of the major leading causes of cancer death worldwide, with increasing incidence, particularly in South Asia [1]. In addition to betel chewing in South Asia, smoking and alcohol consumption are the most common risk factors for OSCC development. Surgery and postoperative radiation therapy are standard procedures for OSCC treatment. Growing studies have also shown that several prognostic factors are associated with poor survival or relapse [2]. Nevertheless, none of the prognostic factors have been proven to be a suitable target for OSCC. Thus, the 5-year overall survival rate has not significantly improved in the last decade, which supports the urgent requirement for suitable therapeutic biomarkers or targets for OSCC.

Kinases play important roles in tumor development and malignancy in various types of cancer. When kinase is constitutively activated resulting from overexpression or mutation or chromosome translocation, it could promote tumorigenesis and drug resistance of cancer cells. For example, epidermal growth factor receptor (*EGFR*) and human epidermal growth factor receptor 2 (*HER2*) are overexpressed in colorectal cancer and breast cancer, respectively. The V599E and V600E mutations in v-raf murine sarcoma viral oncogene homolog B1 (*BRAF*) are observed in various carcinomas [3], while the L858R mutation in *EGFR* is observed in non-small cell lung cancer (NSCLC) cells [4]. The translocation of the *ALK* and *ABL* genes is a major driver for NSCLC and chronic myeloid leukemia, respectively [5,6]. These kinases have been used as targets for cancer therapy in a clinical setting. However, little is known about kinases in OSCC.

Small interfering RNA (siRNA) screening has been used to identify potential targets in diseases, particularly cancer. Kinome-wide screening results have revealed that targeting Mitogen-activated protein kinase kinase kinase 7 (*MAP3K7*) suppressed the proliferation of hepatocellular carcinoma cells in cell culture and tumor xenograft models [7,8]. MAP3K7 is overexpressed in tumor tissues and is associated with poor survival [7]. Moreover, triple-negative breast cancer cells were silenced with the Kinome siRNA library, and it was found that Src might be the major cause of drug resistance. In this study, through high throughput screening with an arrayed kinome siRNA library and The Cancer Genome Atlas (TCGA) analysis, we found that polo-like kinase 1 (*PLK1*) might be a potential target in OSCC.

## 2. Experimental Procedure

### 2.1. Cell Culture, Treatments, and Transfections

**Figure 1:**
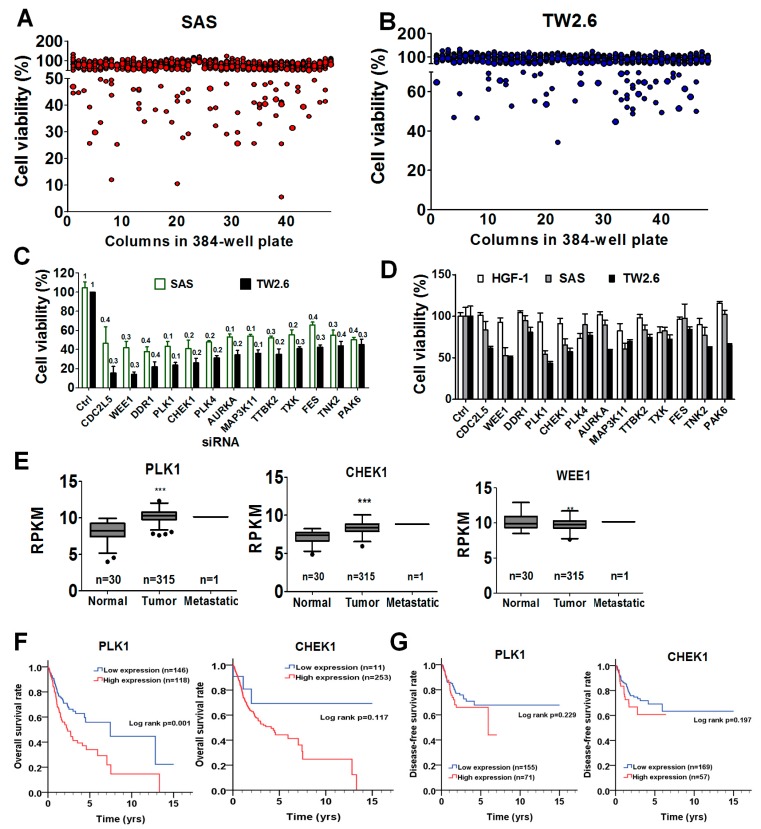
Kinome siRNA (small interfering RNA) library screening for kinases required for OSCC (oral squamous cell carcinoma) cell proliferation. (**A**) SAS and (**B**) TW2.6 cells were seeded and transfected with the arrayed kinome siRNA library (5 nM pooled siRNAs for each gene) for 72 h. Cell viability was measured by a CellTiter-Glo assay (Promega, Madison, WI, USA). (**C**) Cells were transfected with the top 13 ranked siRNAs (as listed) that reduced cell viability in both SAS (white) and TW2.6 (black) cells. The mRNA level in each gene was determined by real-time PCR to confirm the knockdown efficiency of siRNA using scramble siRNA (Ctrl) as normalized controls. The fold change of mRNA level in each siRNA knockdown is indicated above each bar. (**D**) The siRNAs were further counter-assayed with normal human gingival cells (HGF-1). The selective effects of silencing three genes, namely, *PLK1, CHEK1, and WEE1*, are shown. (**E**) The expression of the three genes in OSCC patients is presented as reads per kilobase of transcript per million mapped reads (RPKM) to compare its transcription level between tumor tissues and adjacent normal tissues according to the TCGA (The Cancer Genome Atlas) dataset. * *p* < 0.05, ** *p* < 0.01, *** *p* < 0.001, tumor tissues vs. the adjacent normal tissues. (**F**) Kaplan-Meier plots were used to evaluate the gene association with overall survival in patients with OSCC. (**G**) Kaplan-Meier plots were used to evaluate the gene association with disease-free survival in patients with OSCC.

SAS and TW2.6 cells were cultured in Dulbecco’s Modified Eagle Medium (DMEM) and DMEM/F12 medium supplemented with 10% FBS, 100 μg/mL streptomycin, 100 IU penicillin, and 1% L-glutamine at 37 °C in 5% CO2:95% air. The cells were transfected with 10 nM arrayed Kinome siRNA (709 genes, A30079, Thermo Fisher Scientific, Carlsbad, CA, USA) in 384-well plates using RNAiMAX (13778150; Life Technologies, Carlsbad, CA, USA) for 72 h. The knockdown efficiency of thirteen gene hits, as described in Figure 1C, was verified by real-time PCR. The primer sets for these gene expressions could be provided upon request. Furthermore, the cells were transfected with a siRNA against *PLK1* (L-003290-00-0005; Dharmacon, Lafayette, LA, USA) or an antisense oligonucleotide (ASO) against *PLK1* (ASO-*PLK1*-16: TCATTAAGCAGCTCGT; ASO-*PLK1*-14: CATTAAGCAGCTCG, EXIQON, Vedbaek, Denmark) for at least 48 h. The knockdown efficiency was validated by immunoblotting. Moreover, the cells (2 × 10^6^) were seeded into 6-well culture dishes overnight and treated with the PLK1 kinase inhibitor (BI6727, Selleckchem, Houston, TX, USA).

### 2.2. Western Blot for Protein Expression

Human oral cancer cells were briefly rinsed in PBS and lysed with RIPA buffer (1% NP40, 50 mM Tris-Cl, pH 7.5, 150 mM NaCl, 0.25% sodium deoxycholate, 0.1% SDS, and protease inhibitor cocktail). The cell lysates were resolved by SDS–PAGE (sodium dodecyl sulfate–polyacrylamide gel electrophoresis) and transferred electrophoretically onto nitrocellulose membranes. The membrane was blocked with 5% skim milk and then incubated with the primary antibodies, including anti-PLK (ab17056, Abcam, Eugene, OR, USA), anti-phospho-CCNB1 (ab60986, Abcam, Eugene, OR, USA), and anti-actin (A5441, Sigma-Aldrich, St. Louis, MO, USA), at 4 ℃ overnight. The proteins were probed with an horseradish peroxidase (HRP)-labeled secondary antibody and detected with ECL reagent. The membrane was scanned and analyzed for protein expression with Odyssey Imaging Systems (LI-COR Biosciences, Lincoln, NE, USA).

### 2.3. Clonogenic Assay

Human cancer cells were seeded into six-well plates at a density of 10^3^ cells/well in the presence of inhibitors or the ASO or siRNA against PLK. Cells were then washed and cultured in complete media, and fresh media was provided every 3 days for 2 weeks. The cell colonies were fixed with 2% paraformaldehyde and stained with 20% ethanol containing 0.25% crystal violet at room temperature for 30 min [8]. The stained cells were washed with PBS 3 times to observe colonies. Colonies over 1 mm were counted, and the ratio of the number of colonies formed between cells treated with inhibitors or an ASO against PLK was calculated with Prism 5 (San Diego, CA, USA).

### 2.4. Cell Viability

The cell viability assay was performed using a CellTiter-Glo Luminescent Cell Viability Assay Kit (Promega, Madison, WI, USA). Briefly, 5 × 10^5^ to 7 × 10^5^ cells/mL were cultured in sterile 96-well plates for 24 to 48 h, and then 100 μL of CellTiter-Glo reagent was added to lyse the cells for 10 min. The luminescence signal was measured using a Fluoroskan Ascent FL reader (Thermo Fisher Scientific, Carlsbad, CA, USA).

### 2.5. Flow Cytometry for Cell Cycle Analysis

The knockdown cells were washed with PBS and fixed with 75% ethanol at −20 °C overnight, as previously described [9]. Fixed cells were washed with PBS containing 1% FBS and further stained with propidium iodide (50 μg/mL, Sigma-Aldrich, St. Louis, MO, USA) and RNase A (25 μg/mL) on ice for 30 min. The stained cells were analyzed for cell cycle proportion by using FACScan (Becton Dickinson, San Diego, CA, USA).

### 2.6. Statistical Analysis

The gene expression data and clinical information of OSCC were obtained from the TCGA dataset. The Kruskal-Wallis one-way ANOVA test was used to evaluate the differential gene expression in tumor adjacent normal (TAN) tissues and tumor tissues in OSCC data obtained from the TCGA database. The gene expression levels between TAN tissues and tumor tissues were analyzed by the Wilcoxon signed-rank test. The gene expression level was divided into a high and low group according to receiver operating characteristic (ROC) curve. The Kaplan-Meier method was used to analyze cumulative survival curves, and a survival curve analysis was performed using the log-rank test. The Cox proportional hazards model was used to evaluate the impact of gene expression on survival using factors identified as significant in the univariate analysis as covariates. The association between cell differentiation and the relative gene expression levels in tumor tissues compared with those in the paired TAN tissues of individual OSCC patients with different AJCC (American Joint Committee on Cancer) pathological stages was determined by Fisher’s exact test. A value of *p* < 0.05 was considered statistically significant and has been marked as bold in each table.

## 3. Results

### 3.1. High-Throughput Screening for Potential Targets in OSCC Cells

To determine whether any kinase is crucial for the survival of OSCC cells, human tongue cancer SAS cells and buccal mucosa TW2.6 cells were transfected with a kinome siRNA library for 72 h, and the viable cells were measured with CellTiter-Glo according to the cellular ATP levels (Figure 1A,B). Genes in which cell proliferation was less than 60% in both SAS and TW2.6 cells after knockdown were selected as candidate genes for further validation (Figure 1C). Thirteen genes were selected after screening: *CDC2L5, WEE1, DDR1, PLK1, CHEK1, PLK4, AURKA, MAP3K11, TTBK2, TXK, FES, TNK2, and PAK6*. Human gingival fibroblasts (HGF-1) are normal oral cells and can be used as counter cells to eliminate gene hits that are also required for normal cell physiology (Figure 1D). Silencing *WEE1, PLK1*, or *CHEK1* could also reduce the gene expression in HGF cells. The viability results showed that knockdown of the genes selectively reduced cancer cell viability without affecting normal HGF-1 cells, suggesting that these three genes might be worthy of follow up. We further evaluated the expression of candidate genes in OSCC patients with the TCGA dataset, which consists of corresponding normal tissues from patients (normal, *n* = 30), primary tumors (*n* = 315), and metastatic tumors (*n* = 2) (Figure 1E). The expression of *PLK1* and *CHEK1* was significantly higher in tumor tissues than in normal tissues, whereas WEE1 expression was significantly lower in tumor tissues than that in normal tissues (Figure 1E). Moreover, patients with high *PLK1* expression had shorter overall survival than those with low *PLK1* expression (*p* < 0.001, Figure 1F). However, *CHEK1* expression was not significantly associated with overall survival (*p* = 0.119). The expression of either *PLK1* (*p* = 0.240) or *CHEK1* (*p* = 0.210) had no significant correlation with disease-free survival (Figure 1G).

### 3.2. Genetic Ablation of PLK1-Attenuated Oral Cancer Cell Proliferation

**Figure 2:**
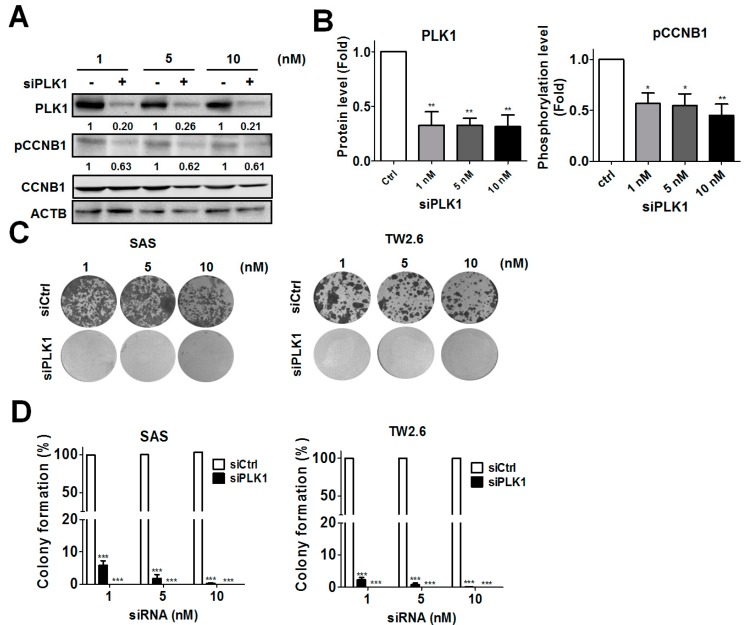
Effects of the siRNA (small interfering RNA) against *PLK1* (polo-like kinase 1) on colony formation in OSCC (oral squamous cell carcinoma) cells. (**A**) Human oral cancer SAS cells were transfected with a siRNA (1, 5, and 10 nM) against *PLK1* kinase for 48 h. The knockdown efficiency of *PLK1* and the phosphorylation of its downstream protein, cyclin B1 (CCNB1), in the cells were examined by immunoblotting. (**B**) The quantitative results for (A) are expressed as the mean ± standard error of the mean (SEM) from three independent experiments. * *p* < 0.05, ** *p* < 0.01 vs. the non-targeting control siRNA (siCtrl). (**C**) SAS and TW2.6 cells were transfected with various concentrations of the siRNA against *PLK1* and cultured for 14 days. The cells were fixed and stained with crystal violet to count the number of colonies formed. (**D**) The quantitative results for (C) are expressed as the mean ± SEM from three independent experiments. *** *p* < 0.001 vs. the non-targeting control siRNA (siCtrl).

PLK1 is reportedly responsible for cyclin B1 (CCNB1) phosphorylation to promote the G_2_-M phase transition in the cell cycle [10]. To further determine the effects of the siRNA against *PLK1* on CCNB1 phosphorylation in oral cancer cells, SAS and TW2.6 cells were transfected with various concentrations of pooled siRNAs against *PLK1*, which target different gene loci from the original siRNA used in the primary screen. A low dose of pooled siRNAs against *PLK1* (1 nM) effectively reduced the PLK1 protein level and the phosphorylation of CCNB1 at Ser 133 (Figure 2A,B). The long-term effects of the siRNA against *PLK1* on the colony formation of oral cancer cells were examined with a clonogenic assay (Figure 2C,D). Silencing *PLK1* with the siRNA significantly suppressed colony formation in both SAS and TW2.6 oral cancer cells.

**Figure 3:**
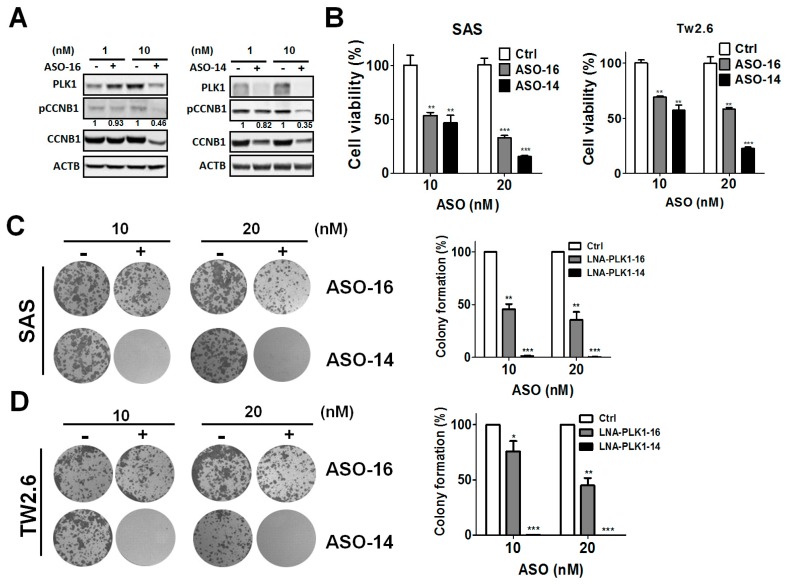
Effects of antisense oligonucleotides against *PLK1* (polo-like kinase 1) on the proliferation of OSCC (oral squamous cell carcinoma) cells. (**A**) Human oral cancer SAS cells were transfected with antisense oligonucleotides (ASO-16 and ASO-14) against *PLK1* for 48 h. The knockdown efficiency of PLK1 and the effects of its downstream protein, cyclin B1 (CCNB1), in the cells were examined by immunoblotting. (**B**) SAS and TW2.6 cells were transfected with ASO-16 or ASO-14 (10 and 20 nM) for 72 h, and cell proliferation was measured with the CellTiter-Glo assay (Promega). The quantitative results for (A) are expressed as the mean ± standard error of the mean (SEM) from three independent experiments., ** *p* < 0.01, *** *p* < 0.001 vs. the non-targeting control siRNA (siCtrl). (**C**) SAS and (**D**) TW2.6 cells were transfected with ASO-16 or ASO-14 against *PLK1* and cultured for 14 days. The cells were fixed and stained with crystal violet to count the number of colonies formed (left panel). The quantitative results are expressed as the mean ± SEM from three independent experiments (right panel). * *p* < 0.05, ** *p* < 0.01, *** *p* < 0.001 vs. the non-targeting control siRNA (siCtrl).

More interestingly, we designed different lengths of locked antisense oligonucleotides against *PLK1* (ASO-*PLK1*-16 or ASO-*PLK1*-14) and examined their effects on the knockdown efficiency of PLK1 and the phosphorylation of CCNB1 (Figure 3A). Although both antisense oligonucleotides at a concentration of 10 nM showed sufficient silencing effects on PLK1, ASO-*PLK1*-14 had better knockdown efficiency on *PLK1* than did ASO-*PLK1*-16 (Figure 3A). Accordingly, CCNB1 phosphorylation was decreased in oral cancer cells. Moreover, ASO-*PLK1* significantly reduced the viability of SAS and TW2.6 cells (Figure 3B). The long-term effects of ASO-*PLK1* on cancer cell growth were inspected with a clonogenic assay (Figure 3C,D). Similar to the knockdown efficiency, the suppressive effects of ASO-*PLK1*-14 on colony formation were stronger than those of ASO-*PLK1*-16. These results indicated that ASO-*PLK1*-14 might be more potent on silencing effects of *PLK1* and growth inhibition in oral cancer cells.

### 3.3. Pharmacological Ablation of PLK1-Attenuated Oral Cancer Cell Proliferation

**Figure 4:**
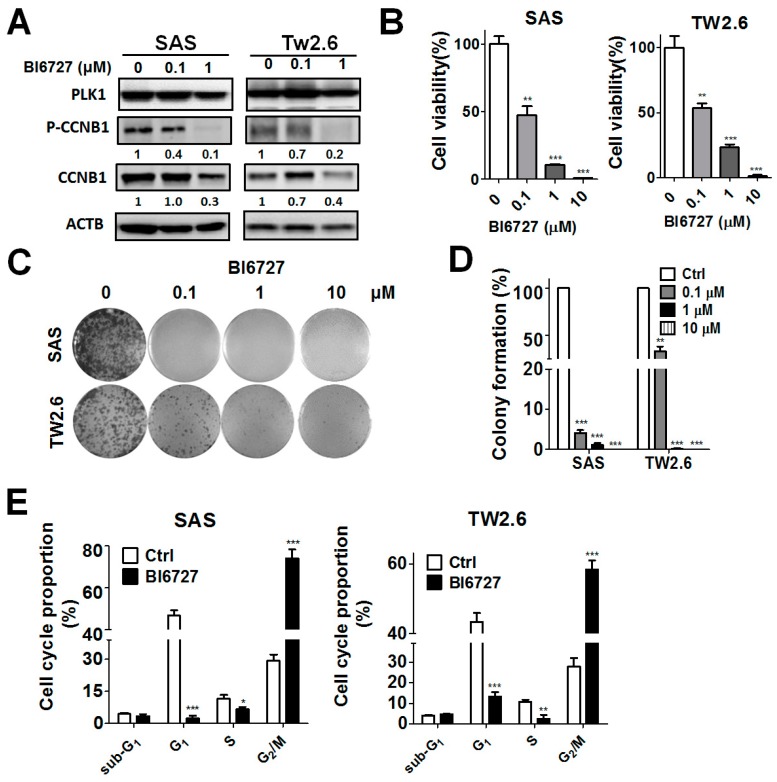
Effects of the PLK1 (polo-like kinase 1) inhibitor on the proliferation of OSCC (oral squamous cell carcinoma) cells. (**A**) Human oral cancer SAS and TW2.6 cells were treated with the PLK1 inhibitor BI6727 for 24 h and harvested for immunoblotting to measure the protein level of PLK1, CCNB1 (cyclin B1), and phosphorylated CCNB1. (**B**) The proliferation of treated cells was assessed with a CellTiter-Glo assay (Promega). The quantitative results are expressed as the mean ± standard error of the mean (SEM) from three independent experiments. ** *p* < 0.01, *** *p* < 0.001 vs. the non-targeting control siRNA (siCtrl). (**C**) The cells were treated with various concentrations of BI6727 to observe colony formation with crystal violet. (**D**) The quantitative results are expressed as the mean ± SEM from three independent experiments. * *p* < 0.05, ** *p* < 0.01, *** *p* < 0.001 vs. the non-targeting control siRNA (siCtrl). (**E**) The cells were treated with BI6727 (1 µM) for 24 h and harvested for cell cycle analysis with a flow cytometer. The proportion of cells in each phase of the cell cycle was quantitated with FlowJo software (BD biosciences, Ashland, OR, USA). * *p* < 0.05, ** *p* < 0.01, *** *p* < 0.001 vs. the cells treated with dimethylsulfoxide (DMSO (Ctrl)).

To determine whether the kinase activity of PLK1 is involved in cancer cell proliferation, oral cancer cells were treated with the PLK1-specific inhibitor BI6727 to examine the CCNB1 expression/phosphorylation and cell proliferation (Figure 4). BI6727 reduced CCNB1 expression and phosphorylation in oral cancer cells (Figure 4A). BI6727 treatment inhibited cell viability and colony formation in SAS and TW2.6 cells in a dose-dependent manner (Figure 4B–D). Besides, PLK1 inhibitors may induce a cell cycle arrest at the G_2_-M phase and apoptosis in cancer cells [11]. We further investigated the effects of BI6727 in the cell cycle of SAS and TW2.6 oral cancer cells (Figure 4E). BI6727 largely caused a G_2_-M phase arrest in cell cycle progression, whereas it did not affect sub-G_1_ phase, suggesting that PLK1 inhibition-reduced cell proliferation might mainly occur through a cell cycle arrest at G_2_-M phase in oral cancer cells.

### 3.4. The Association of PLK1 with Prognosis According to the Demographical and Clinicopathological Features of OSCC Patients

**Figure 5:**
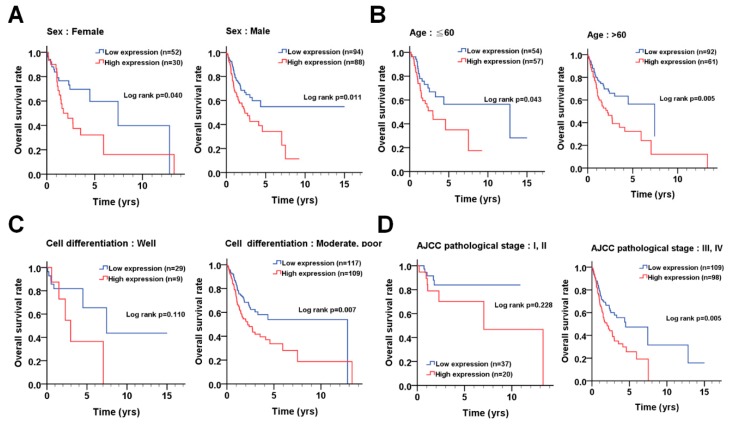
The association of *PLK1* (polo-like kinase 1) gene expression with overall survival in certain groups of OSCC (oral squamous cell carcinoma) patients. (**A**) Kaplan-Meier plots were used for the stratified analysis to determine the association of *PLK1* expression with survival based on sex, (**B**) age (60 years old), (**C**) differentiation, and (**D**) AJCC (American Joint Committee on Cancer) pathological stages. The cutoff values for the high or low expression of *PLK1* in tumor tissues were based on the receiver operating characteristic (ROC) curve. The gene expression data and clinical information of OSCC are obtained from the TCGA dataset.

We observed that *PLK1* expression was higher in tumor tissues than that in adjacent normal tissues of OSCC patients (Figure 1). The higher *PLK1* expression was also correlated with shorter overall survival of OSCC patients (Figure 1). To evaluate the detailed association between *PLK1* expression and OSCC, *PLK1* expression was analyzed in OSCC patients with different demographical characteristics and pathological outcomes (Table 1). *PLK1* expression was elevated in tumor tissues with moderate and poor differentiation compared to that in tumor tissues with good differentiation. We stratified the patients into different groups to examine the impact of *PLK1* expression on overall survival according to the demographical and clinicopathological features of the patients (Figure 5). The Kaplan-Meier analysis showed worse effects of *PLK1* expression on overall survival in patients with certain demographical and clinicopathological features (Figure 5), including sex (female: *p* = 0.040; male: *p* = 0.011), age (≤60: *p* = 0.043; >60: *p* = 0.005), moderate/poor differentiation (*p* = 0.007), and advanced AJCC pathological stages (*p* = 0.005). Moreover, the results were further adjusted by cell differentiation and AJCC pathological stage (Table 2). High *PLK1* expression was significantly associated with poor overall survival in males (Table 2, adjusted hazard ratio (AHR) = 1.64, *p* = 0.034) and elderly (>60 years: AHR = 1.64, *p* = 0.017) patients. High *PLK1* expression was correlated with a worse prognosis in tumor tissues with both good (AHR = 4.32, *p* = 0.026) and moderate/poor (AHR = 1.57, *p* = 0.026) differentiation (Table 2). Moreover, high *PLK1* expression was associated with unfavorable overall survival in patients with an advanced pathological stage (Table 2, AHR = 1.67, *p* = 0.014), whereas it was correlated with unfavorable overall survival in patients with early T stage (T1 and T2, AHR = 2.51, *p* = 0.022) and in tumors without lymph node invasion (N0, AHR = 2.24, *p* = 0.023). High *PLK1* expression was also associated with unfavorable overall survival in OSCC patients with (AHR = 2.12, *p* = 0.017) or without (AHR = 1.95, *p* = 0.029) postoperative radiation therapy. Nevertheless, the stratification results showed that *PLK1* expression was not associated with disease-free survival in any patient groups based on clinicopathological features (Table 3). These results suggested that *PLK1* expression might be involved in tumor progression and survival in OSCC patients, depending on demographical and clinicopathological features.

### 3.5. The Correlation of PLK1 and CCNB1 with Prognosis in Patients with OSCC

**Figure 6:**
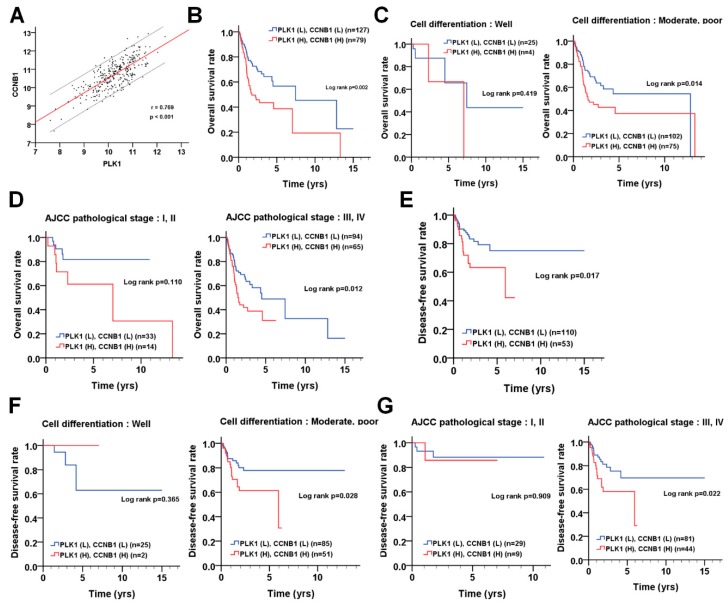
The association of *PLK1* (polo-like kinase 1) and *CCNB1* (cyclin B1) with overall survival and disease-free survival in OSCC (oral squamous cell carcinoma) patients. (**A**) The correlation between *PLK1* and *CCNB1* was estimated by Spearman’s rank correlation coefficient. (**B**) The association of the coexpression levels of *PLK1* and *CCNB1* with overall survival in OSCC patients was analyzed with a Kaplan-Meier curve. The association of *PLK1* and *CCNB1* coexpression with overall survival was further stratified by (**C**) differentiation and (**D**) AJCC (American Joint Committee on Cancer) pathological stage. (**E**) The association of the coexpression levels of *PLK1* and *CCNB1* with disease-free survival in OSCC patients was analyzed with a Kaplan-Meier curve. The association of *PLK1* and *CCNB1* coexpression with disease-free survival was further stratified by (**F**) differentiation and (**G**) AJCC pathological stage. The cutoff values for the high or low expression of *PLK1* in tumor tissues were based on the receiver operating characteristic (ROC) curve. The gene expression data and clinical information of OSCC are obtained from the TCGA dataset.

In addition to the phosphorylation effects of PLK1 on CCNB1, PLK1 binds to the DNA-binding domain of tumor protein p53 (TP53) to block its transcriptional activity, which may induce CCNB1 gene expression [12]. The knockdown of *PLK1* indeed diminished CCNB1 expression in OSCC cells. To determine whether *PLK1* expression is correlated with *CCNB1* expression in patients with OSCC, Pearson’s correlation was used to analyze gene expression in OSCC data from the TCGA dataset. Interestingly, *PLK1* expression was highly correlated with *CCNB1* expression (Figure 6A, r = 0.769, *p* < 0.001). The Kaplan-Meier survival curve showed that the high coexpression of *PLK1* and *CCNB1* was correlated with shorter overall survival (Figure 6B, *p* = 0.002), particularly in cells with moderate/poor differentiation (Figure 6C, *p* = 0.014) and advanced pathological stages (Figure 6D, *p* = 0.012). The association of high *PLK1* and *CCNB1* coexpression with disease-free survival is shown in Figure 6E (*p* = 0.017). Its unfavorable impacts on disease-free survival in certain types of OSCC patients were also observed, such as those with moderate/poor differentiation (Figure 6F, *p* = 0.028) and advanced pathological stages (Figure 6G, *p* = 0.022). Besides, the association of the coexpression level of *PLK1* and *CCNB1* in overall survival and disease-free survival was analyzed with univariate and multivariate Cox proportional hazards models (Table 4). The patients with high coexpression levels of *PLK1* and *CCNB1* had worse overall survival (Table 4, crude hazard ratio (CHR) = 1.60, *p* = 0.016, AHR = 1.98, *p* = 0.002). These results implied that the coexpression of *PLK1* and *CCNB1* might be a potential diagnostic marker for OSCC.

## 4. Discussion

To date, there is almost no targeted therapy for patients with OSCC due to limited targets reported thus far. In this study, we employed a kinome siRNA library to screen potential oncogenic kinases required for the proliferation of OSCC cells and reported the following findings. First, silencing *PLK1* with either a siRNA or ASO attenuated CCNB1 phosphorylation and blocked the proliferation of OSCC cells. Second, the PLK1 inhibitor diminished the expression/phosphorylation of CCNB1 and arrested the cell cycle at the G_2_-M phase. Third, the TCGA data analysis indicated that *PLK1* expression was higher in tumor tissues than in adjacent normal tissues. High *PLK1* expression was also associated with worse overall survival in OSCC patients with certain demographical and pathological features. Fourth, *PLK1* expression was highly correlated with *CCNB1* expression in OSCC patients. The coexpression of *PLK1* and *CCNB1* was significantly correlated with poor overall survival and disease-free survival. Thus, our results may suggest that coexpression of *PLK1-CCNB1* is a good biomarker for patients with OSCC.

Synthetic nucleotides, including siRNAs and ASOs, have been used to knock down gene expression in a wide variety of experiments and clinical trials [13]. The siRNA is delivered as a double strand, and the passenger strand is removed by the argonaute of the RNA-induced silencing complex (RISC). The guide strand then primes the mRNA sequence, as ASO does for gene silencing. The specificity and efficacy of the siRNA depend on the sequence and chemical modifications [14]. Pooled siRNAs may reduce off-target effects due to the limited amount of every single siRNA. Thus, we began our screen with a pooled siRNA library (Thermo Fisher Scientific, Carlsbad, CA, USA) to identify candidate genes essential for the proliferation of OSCC cells. The top-ranked gene, *PLK1*, was further confirmed with different pooled siRNAs (Dharmacon, Lafayette, LO, USA) that contain different sequences and chemical modifications. Both pooled siRNAs against *PLK1* efficiently blocked the proliferation of OSCC cells. Similar to a siRNA, an ASO-generated DNA-RNA hybrid can be degraded by RNase H. Also, an ASO may decrease gene expression via the attenuation of translation and RNA splicing [13]. The ASO against *PLK1* also diminished the growth of OSCC cells, particularly in the short gapmer with eight bases in the central gap (ASO-*PLK1*-14). Our present study supports the notion that *PLK1* is an essential gene for OSCC proliferation.

Our kinome siRNA library screening results showed that *PLK1, CHEK1*, and *WEE1* were the top three selective genes required for the growth of OSCC cells. PLK1 expression is gradually increased and peaks at the M phase of the cell cycle, which activates CDC25, a phosphatase and activator of CDK1, to promote cell cycle progression at the G2-M phase [10]. In contrast to PLK1, WEE1 and CHEK1 are negative regulators in the G2-M phase transition. WEE1 is a tyrosine kinase that inhibits CDK1 by phosphorylating it at Tyr 15 and 14, which in turn arrests the cell cycle at the G_2_ phase in cells during DNA damage [15]. WEE1 is also involved in homologous recombination DNA repair [15]. Moreover, CHEK1 is a serine/threonine kinase and a major downstream regulator of ataxia telangiectasia-mutated and Rad-3-related (ATR) for cell cycle arrest at the G2-M phase transition in cells in response to DNA damage [16]. CHEK1 phosphorylates Ser216 of CDC25 and triggers the proteasomal degradation of CDC25 [17]. Besides, CHEK1 phosphorylates PLK1 to promote its degradation in the proteasome, whereas CHEK1 can phosphorylate and activate WEE1 for cell cycle arrest at the G2-M phase [17]. Although our screening platform was based on two OSCC cell lines, and the functions of these three selective genes in G2-M phase may be different, our results implied that the G2-M phase transition might play a crucial role in OSCC cell proliferation. Nevertheless, the detailed mechanisms by which these genes regulate each other in OSCC cells under normal physiological conditions require further study.

Regarding the roles of the gene hits in cancer, *PLK1* is overexpressed in numerous types of cancer, including lung cancer, breast cancer, hepatocellular carcinoma, head and neck cancer, cholangiocarcinoma, and uterine corpus endometrial carcinoma [10]. WEE1 is highly expressed in brain cancer, breast cancer, and melanoma [18], while CHEK1 expression is higher in the tumor tissues of breast cancer, colon cancer, and liver cancer [17]. The PLK1 inhibitor BI2536 and its derivative BI6727 inhibit tumor growth in vitro and in the clinical setting [19,20], suggesting that a PLK1 inhibitor could be a monotherapy for cancer treatment. In contrast, targeting WEE1 or CHEK1 sensitizes cancer cells to DNA-damaging drugs [21,22], supporting WEE1 and CHEK1 as potential targets and chemosensitizers for cancer therapy. However, our results showed that WEE1 expression was lower in tumor tissues than in adjacent normal tissues and that CHEK1 was not associated with shorter survival. Thus, the roles of WEE1 and CHEK1 in OSCC might need further verification.

Our current study showed that *PLK1* expression was associated with tumorigenesis and the poor prognosis of OSCC patients. The siRNA against *PLK1* selectively inhibited OSCC cell proliferation, implying that *PLK1* could be a potential target for OSCC. Additionally, *PLK1* expression is much higher in tumor tissues than in adjacent normal tissues in certain types of cancer, such as lung cancer (20.8-fold), cholangiocarcinoma (24.3-fold), and uterine corpus endometrial carcinoma (21.3-fold) [10]. Its expression is also higher in head and neck squamous cell carcinoma (4.2-fold). High *PLK1* expression is associated with poor prognosis in head and neck squamous cell carcinoma [23]. Moreover, several PLK1 inhibitors have been tested in clinical trials for advanced solid tumors [24]. Our results indicated that a siRNA or ASO against *PLK1* suppressed the proliferation of OSCC cells. These results suggested that the siRNA or ASO against *PLK1* used in our study might be suitable for testing its efficacy in the cancer types mentioned above.

## 5. Conclusions

In conclusion, PLK1 is required for the G_2_-M phase transition in OSCC cells, likely through CCNB1 expression/phosphorylation. PLK1 expression or its coexpression with CCNB1 was highly correlated with a poor prognosis, particularly in OSCC patients with certain stages, supporting PLK1 could be a potential diagnostic marker or therapeutic target for OSCC patients in the future.

## Figures and Tables

**Table 1:** Association of *PLK1* expressions with clinicopathologic outcomes in patients with OSCC (oral squamous cell carcinoma).

Variable	No. (%)	*PLK1*		
		Mean ± SD	Median	*p*-Value
Sex				
Female	83 (31.1)	10.15 ± 0.76	10.19	0.123 *
Male	184 (68.9)	10.30 ± 0.75	10.36	
Age, y				
≦40	10 (3.8)	10.03 ± 0.85	10.20	0.211 ^†^
41–50	33(12.4)	10.31 ± 0.60	10.38	
51–60	70 (26.3)	10.39 ± 0.63	10.46	
>60	153 (57.6)	10.18 ± 0.82	10.21	
Cell differentiation				
Well	38 (14.2)	9.89 ± 0.74ab	9.97	**0.004** ^†^
Moderate	177 (66.3)	10.30 ± 0.73a	10.29	
Poor	52 (19.5)	10.36 ± 0.78b	10.45	
AJCC pathological stage				
I	18 (6.7)	9.89 ± 0.71	9.91	0.168 ^†^
II	40(15.0)	10.21 ± 0.79	10.25	
III	55 (20.6)	10.24 ± 0.78	10.26	
IV	154(57.7)	10.31 ± 0.73	10.33	
T classification				
T1	27 (10.1)	9.95 ± 0.71	9.92	0.093 ^†^
T2	80 (30.0)	10.23 ± 0.77	10.25	
T3	57 (21.3)	10.24 ± 0.70	10.26	
T4	103 (38.6)	10.36 ± 0.76	10.41	
N classification				
N0	119 (44.6)	10.20 ± 0.73	10.24	0.647 ^§^
N1	46 (17.2)	10.27 ± 0.97	10.33	
N2	99 (37.1)	10.30 ± 0.66	10.31	
N3	3 (1.1)	10.67 ± 0.87	11.06	

The gene expression data and clinical information of OSCC are obtained from the TCGA (The Cancer Genome Atlas) dataset. Abbreviations: SD, standard deviation; SCC, squamous cell carcinoma; AJCC, American Joint Committee on Cancer. * *p*-values were estimated by student’s t-test. ^†^ *p*-values were estimated by one-way ANOVA test. ^§^ *p*-values were estimated by Kruskal-Wallis one-way ANOVA test. ^†^ *p*-values were estimated by Mann-Whitney U test.

**Table 2:** Impact of *PLK1* expression levels on overall survival by the different demographic and clinicopathologic factors with OSCC.

Variable	*PLK1*	No. (%)	CHR (95% CI)	*p*-Value *	AHR (95% CI)	*p*-Value ^†^
Sex						
Female	Low	52 (63.4)	1.00		1.00	
	High	30 (36.6)	1.98 (1.02–3.87)	**0.044**	2.06 (0.97–4.39)	0.060 ^a^
Male	Low	94 (51.6)	1.00		1.00	
	High	88 (48.4)	1.78 (1.13–2.79)	**0.012**	1.64 (1.04–2.59)	**0.034 ^a^**
Age, yrs						
≦60	Low	54 (48.6)	1.00		1.00	
	High	57 (51.4)	1.85 (1.01–3.38)	**0.046**	1.76 (0.96–3.22)	0.069 ^a^
>60	Low	92 (60.1)	1.00		1.00	
	High	61 (39.9)	1.97 (1.22–3.18)	**0.006**	1.86 (1.12–3.11)	**0.017 ^a^**
Cell differentiation						
Well	Low	29 (76.3)	1.00		1.00	
	High	9 (23.7)	2.54 (0.78–8.35)	0.124	4.32 (1.19–15.70)	**0.026 ^b^**
Moderate, poor	Low	117 (51.8)	1.00		1.00	
	High	109 (48.2)	1.71 (1.15–2.55)	**0.008**	1.57 (1.06–2.34)	**0.026 ^b^**
AJCC pathological stage						
I, II	Low	37 (64.9)	1.00		1.00	
	High	20 (35.1)	2.06 (0.62–6.84)	0.237	2.09 (0.63–6.96)	0.229 ^c^
III, IV	Low	109 (52.7)	1.00		1.00	
	High	98 (47.3)	1.75 (1.18–2.60)	**0.005**	1.67 (1.11–2.51)	**0.014 ^c^**
T classification						
T1, T2	Low	65 (62.5)	1.00		1.00	
	High	39 (37.5)	2.48 (1.14–5.43)	**0.023**	2.51 (1.14–5.50)	**0.022 ^d^**
T3, T4	Low	81 (50.6)	1.00		1.00	
	High	79 (49.4)	1.50 (0.98–2.29)	0.063	1.42 (0.91–2.21)	0.118 ^d^
N classification						
N0	Low	70 (59.3)	1.00		1.00	
	High	48 (40.7)	2.32 (1.21–4.45)	**0.011**	2.24 (1.12–4.49)	**0.023 ^e^**
N1, N2, N3	Low	76 (52.1)	1.00		1.00	
	High	70 (47.9)	1.60 (1.01–2.53)	**0.045**	1.37 (0.86–2.19)	0.187 ^e^
Postoperative RT						
No	Low	56 (56.6)	1.00		1.00	
	High	43 (43.4)	2.54 (1.40–4.64)	**0.002**	2.12 (1.14–3.92)	**0.017 ^a^**
Yes	Low	73 (52.5)	1.00		1.00	
	High	66 (47.5)	2.08 (1.17–3.73)	**0.013**	1.95 (1.07–3.54)	**0.029 ^a^**

The gene expression data and clinical information of OSCC are obtained from the TCGA dataset. Abbreviations: SCC, squamous cell carcinoma; CHR, crude hazard ratio; CI, confidence interval; AHR, adjusted hazard ratio; AJCC, American Joint Committee on Cancer; RT, radiotherapy. * *p*-values were estimated by Cox’s regression. ^†^ *p*-values were estimated by multivariate Cox’s regression. ^a^ Adjusted for cell differentiation (moderate + poor vs. well) and AJCC pathological stage (stage III + IV vs. stage I + II). ^b^ Adjusted for AJCC pathological stage (stage III + IV vs. stage I + II). ^c^ Adjusted for cell differentiation (moderate + poor vs. well). ^d^ Adjusted for cell differentiation (moderate + poor vs. well) and N classification (N1, N2 vs. N0). ^e^ Adjusted for cell differentiation (moderate + poor vs. well) and T classification (T3, T4 vs. T1 + T2).

**Table 3:** Impact of *PLK1* expression levels on disease-free survival by the different demographic and clinicopathologic factors with OSCC.

Variable	*PLK1*	No. (%)	CHR (95% CI)	*p*-Value *	AHR (95% CI)	*p*-Value ^†^
Sex						
Female	Low	56 (76.7)	1.00		1.00	
	High	17 (23.3)	1.87 (0.85–4.13)	0.120	1.63 (0.70–3.82)	0.257 ^a^
Male	Low	99 (64.7)	1.00		1.00	
	High	54 (35.3)	1.24 (0.73–2.12)	0.426	1.09 (0.63–1.87)	0.765 ^a^
Age, yrs						
≦60	Low	61 (64.9)	1.00		1.00	
	High	33 (35.1)	1.22 (0.61–2.46)	0.571	1.11 (0.55–2.25)	0.766 ^a^
>60	Low	94 (71.2)	1.00		1.00	
	High	38 (28.8)	1.54 (0.87–2.73)	0.137	1.31 (0.72–2.39)	0.374 ^a^
Cell differentiation						
Well	Low	32 (91.4)	1.00		1.00	
	High	3 (8.6)	1.33 (0.27–6.65)	0.731	3.56 (0.63–20.25)	0.153 ^b^
Moderate, poor	Low	123 (64.4)	1.00		1.00	
	High	68 (35.6)	1.34 (0.84–2.13)	0.222	1.15 (0.72–1.85)	0.557 ^b^
AJCC pathological stage						
I, II	Low	41 (78.8)	1.00		1.00	
	High	11 (21.2)	1.98 (0.49–7.95)	0.334	2.24 (0.55–9.17)	0.262 ^c^
III, IV	Low	114 65.5)	1.00		1.00	
	High	60 (34.5)	1.20 (0.76–1.92)	0.435	1.14 (0.71–1.85)	0.582 ^c^
T classification						
T1, T2	Low	71 (74.0)	1.00		1.00	
	High	25 (26.0)	1.61 (0.64–4.04)	0.310	1.40 (0.54–3.60)	0.489 ^d^
T3, T4	Low	84 (64.6)	1.00		1.00	
	High	46 (35.4)	1.20 (0.72–1.99)	0.480	1.16 (0.69–1.95)	0.580 ^d^
N classification						
N0	Low	80 (74.8)	1.00		1.00	
	High	27 (25.2)	1.35 (0.64–2.86)	0.428	1.11 (0.52–2.40)	0.785 ^e^
N1, N2	Low	75 (63.0)	1.00		1.00	
	High	44 (37.0)	1.31 (0.75–2.28)	0.342	1.23 (0.70–2.17)	0.466 ^e^
Postoperative RT						
No	Low	62 (70.5)	1.00		1.00	
	High	26 (29.5)	1.75 (0.93–3.29)	0.084	1.28 (0.66–2.46)	0.467 ^a^
Yes	Low	85 (66.4)	1.00		1.00	
	High	43 (33.6)	1.51 (0.79–2.88)	0.214	1.43 (0.74–2.78)	0.288 ^a^

The gene expression data and clinical information of OSCC are obtained from the TCGA dataset (https://cancergenome.nih.gov/). Abbreviations: SCC, squamous cell carcinoma; CHR, crude hazard ratio; CI, confidence interval; AHR, adjusted hazard ratio; AJCC, American Joint Committee on Cancer; RT, radiotherapy. * *p*-values were estimated by Cox’s regression. ^†^ *p*-values were estimated by multivariate Cox’s regression. ^a^ Adjusted for cell differentiation (moderate + poor vs. well) and AJCC pathological stage (stage III + IV vs. stage I + II). ^b^ Adjusted for AJCC pathological stage (stage III + IV vs. stage I + II). ^c^ Adjusted for cell differentiation (moderate + poor vs. well). ^d^ Adjusted for cell differentiation (moderate + poor vs. well) and N classification (N1, N2 vs. N0). ^e^ Adjusted for cell differentiation (moderate + poor vs. well) and T classification (T3, T4 vs. T1+T2).

**Table 4:** Impact of expression levels of *PLK1* and *CCNB1* on survival by the different demographic and clinicopathologic factors with OSCC.

Variable		No. (%)	CHR (95% CI)	*p*-Value	AHR (95% CI)	*p*-Value
**Overall survival**						
*PLK1*	Low	146 (55.3)	1.00		1.00	
	High	118 (44.7)	1.87 (1.29–2.71)	0.001 *	1.73 (1.18–2.53)	**0.005 ^a^**
*CCNB1*	Low	166 (62.9)	1.00		1.00	
	High	98 (37.1)	1.53 (1.06–2.22)	0.024 *	1.50 (1.03–2.18)	**0.035 ^a^**
*PLK1* (L) *CCNB1* (L)		127 (48.1)	1.00		1.00	
either		58 (22.0)	1.33 (0.88–2.01)	0.172 *	1.79 (1.12–2.85)	**0.015 ^†^**
*PLK1* (H) *CCNB1* (H)		79(29.9)	1.60 (1.09–2.33)	0.016 *	1.98 (1.29–3.05)	**0.002 ^†^**
**Disease-free survival**						
*PLK1*	Low	155 (68.6)	1.00		1.00	
	High	71 (31.4)	1.39 (0.81–2.40)	0.231 *	1.19 (0.68–2.08)	0.540 ^a^
*CCNB1*	Low	128 (56.6)	1.00		1.00	
	High	98 (43.4)	2.30 (1.34–3.95)	0.003 *	2.22 (1.28–3.86)	**0.005 ^a^**
*PLK1* (L) *CCNB1* (L)		110 (48.7)	1.00		1.00	
either		63 (27.9)	1.69 (0.97–2.93)	0.063 *	2.30 (1.21–4.36)	**0.011 ^†^**
*PLK1* (H) *CCNB1* (H)		53 (23.5)	1.55 (0.87–2.74)	0.136 *	2.20 (1.13–4.27)	**0.020 ^†^**

The gene expression data and clinical information of OSCC are obtained from the TCGA dataset. Abbreviations: CHR, crude hazard ratio; CI, confidence interval; AHR, adjusted hazard ratio; * *p*-values were estimated by Cox’s regression; ^†^ *p*-values were estimated by multivariate Cox’s regression. ^a^ Adjusted for cell differentiation (moderate + poor vs. well) and AJCC pathological stage (stage III + IV vs. stage I + II) by multivariate Cox’s regression. CCNB1, cyclin B1; L, Low; H, High.
